# ﻿Two new species and new host and distribution records of *Gnathia* Leach, 1814 (Crustacea, Isopoda, Gnathiidae) from Western Australia and the Great Barrier Reef, Australia

**DOI:** 10.3897/zookeys.1193.116538

**Published:** 2024-03-05

**Authors:** Yuzo Ota, Anja Erasmus, Alexandra S. Grutter, Nico J. Smit

**Affiliations:** 1 San‘in Kaigan Geopark Museum of the Earth and Sea, 1794-4, Makidani, Iwami-cho, Iwami-gun, Tottori 681-0001, Japan San‘in Kaigan Geopark Museum of the Earth and Sea Tottori Japan; 2 Water Research Group, Unit for Environmental Sciences and Management, North-West University, Private Bag X6001, Potchefstroom, 2520, South Africa North-West University Potchefstroom South Africa; 3 School of the Environment, The University of Queensland, St. Lucia, Queensland 4072, Australia The University of Queensland Queensland Australia

**Keywords:** Coral reefs, elasmobranchs, Heron Island, Lizard Island, marine fish parasites, Rottnest Island, taxonomy

## Abstract

*Gnathiaantennacrassa***sp. nov.** from seagrass beds off Rottnest Island, Western Australia is the first record of any gnathiid from the entirety of Western Australia; the male can be distinguished from congeners by the stout peduncular articles of the antenna. *Gnathiataurus***sp. nov.** is described from two adult specimens reared from praniza larvae found infecting elasmobranch fishes at Heron Island, southern Great Barrier Reef; the males can be distinguished from all congeners by the dorsally strongly elongate mandibles and smoothly rounded mediofrontal process on the anterior part of cephalosome. Gnathiaaff.maculosa Ota & Hirose, 2009 is recorded from Australia, together with further records of *G.trimaculata* Coetzee, Smit, Grutter & Davies, 2009 and *G.grandilaris* Coetzee, Smit, Grutter & Davies, 2008, all from elasmobranch fishes.

## ﻿Introduction

The isopod family Gnathiidae Leach, 1814, exhibits a biphasic lifecycle characterised by morphological differentiation among its larvae (juveniles) as well as between adult males and adult females. Gnathiid larvae are temporary ectoparasites of marine teleosts and elasmobranchs. In contrast, the adult stage is non-parasitic and reproduces in benthic substrates (Smit and Davies 2004; Tanaka 2007).

The Gnathiidae includes 12 genera and approximately 240 species worldwide (Boyko et al. 2023). The Australia gnathiids are represented by seven genera and 60 species, which have almost exclusively been described from the eastern coasts of Australia from South Australia to Queensland. The majority of species in Australia were collected from benthic substrata (Haswell 1884; Beddard 1886; Hale 1924; Monod 1926; Cals, 1973; Seed 1979; Holdich and Harrison 1980; Cohen and Poore 1994; Svavarsson and Bruce 2012, 2019), while several studies have described species from adult specimens reared from juveniles collected from host fishes (Coetzee et al. 2008, 2009; Ferreira et al. 2009, 2010; Farquharson et al. 2012).

Two new species are here described, *Gnathiaantennacrassa* sp. nov. from Rottnest Island, southern Western Australia and *Gnathiataurus* sp. nov. from Heron Island, southern Great Barrier Reef (GBR). Additionally, we report Gnathiaaff.maculosa Ota & Hirose, 2009, a new record for Australia and provide new host and distribution records of two other *Gnathia* species from the GBR.

## ﻿Materials and methods

Larval isopod samples from the GBR were collected from five elasmobranch species as part of parasitological research on elasmobranchs conducted during the 1990s (Great Barrier Reef Marine Park Permit no. G96/543). These samples were reared to adults in vials containing seawater. Some of these larvae moulted into adult males suitable for species description.

Adult male specimens were preserved in 70% ethanol, and total length measured between the tip of the mandibles and posterior margin of pleotelson. Additionally, their body length was measured between the anterior margins of the frontal processes and posterior margin of pleotelson. Specimens were cleaned using a fine hair of saturated polyester resin and dissected with sharpened tungsten needles. The appendages were removed from the body and then mounted in CMCP-10 high-viscosity medium (Polyscience, Warrington, PA, USA). Observations were conducted using a phase-contrast light microscope, and drawings were made using a camera lucida.

For scanning electron microscope (SEM) examination, one specimen was dehydrated in 99% ethanol for a day and air-dried. The dried specimen was mounted on brass SEM stubs using double-sided conductive tape, followed by sputter-coating with platinum, and then photographed using a Hitachi SU3900 SEM. New descriptions were prepared in DEscriptive Language for TAxonomy (DELTA; Dallwitz 2018) using a modified *Gnathia* character set (Erasmus et al. 2023). Descriptive terminology follows Smit and Davies (2004) for setal classification and Cohen and Poore (1994) for the male morphology. Most literature in Gnathiidae treats the fourth article of antennule as the first article of the flagellum, but this article has penicillate seta characteristic of the peduncles, so it is treated as the fourth article of the peduncles. The scientific names used in the host fish records follow FishBase (Froese and Pauly 2023).

Abbreviations: **BL**—body length; **GBR**—Great Barrier Reef, Queensland; **QM**—Queensland Museum, Brisbane; **SEM**—scanning electron microscope; **TL**—total body length; **WAM**—Western Australian Museum, Perth.

## ﻿Taxonomy

**Suborder Cymothoida Wägele, 1989**

**Superfamily Cymothooidea Leach, 1814**

**Family Gnathiidae Leach, 1814**

### ﻿ Gnathia

Taxon classificationAnimaliaIsopodaGnathiidae

Leach, 1814

FA328D6C-9D56-5E5B-A77D-F6A81AFAE310

#### Type species.

*Gnathiatermitoides* Leach, 1814 (= *Cancermaxillaris* Montagu, 1804); by monotypy (Cohen and Poore 1994); type locality: south coast of Devon, Cornwall Peninsula, south west England.

### ﻿ Gnathia antennacrassa sp. nov.

Taxon classificationAnimaliaIsopodaGnathiidae

DB278FC0-C29C-59D5-87D3-43D9D6B7AE86

https://zoobank.org/DB30A1C7-2DCE-458B-920C-56AD365203EE

Figs 1 , 2 , 3

#### Diagnosis.

Anterior part of body (cephalosome and pereonites 1–4) not densely covered by tubercles; frontal margin with serrated triangular mediofrontal process and two superior frontolateral processes; paraocular ornamentation not developed; pereonite 1 not reaching lateral margins of cephalon; pereonites 4–6 with two lateral lobes; epimera of pleonites 1–5 not prominent; pleotelson 0.8 × shorter than its anterior width; lateral side of pleotelson sinuate; maximum width of peduncle article of antenna 3.2 × maximum width of flagellar article; article 1 of pylopod with two areolae; appendix masculina of pleopod 2 0.8 × as long as endopod; endopod of uropodal rami extend beyond apex of pleotelson; exopod of uropodal rami almost apex of pleotelson.

#### Material examined.

***Holotype*.** Australia • 1♂ (2.4 mm TL, 1.9 mm BL, dissected); sandy substrata of seagrass *Amphibolisgriffithii* (J.M. Black) Hartog, 1970 patch bed surrounded by the seagrass *Posidoniasinuosa* Cambridge & Kuo, 1979 bed, 5 m depth, Thomson Bay, Rottnest Island, Western Australia (32°00'S, 115°32.5'E), 18 January 1996, Hiroshi Mukai leg. (WAM C-79675).

#### Type locality.

Thomson Bay, Rottnest Island, Western Australia (32°00'S, 115°32.5'E).

#### Description.

**Figure 1.**
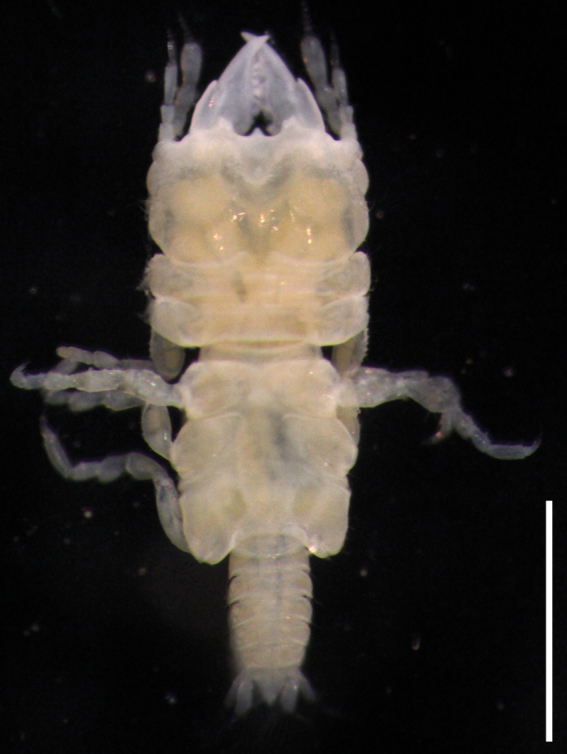
Photograph of fixed *Gnathiaantennacrassa* sp. nov. (holotype, WAM C-79675). Scale bar: 1 mm.

**Figure 2.**
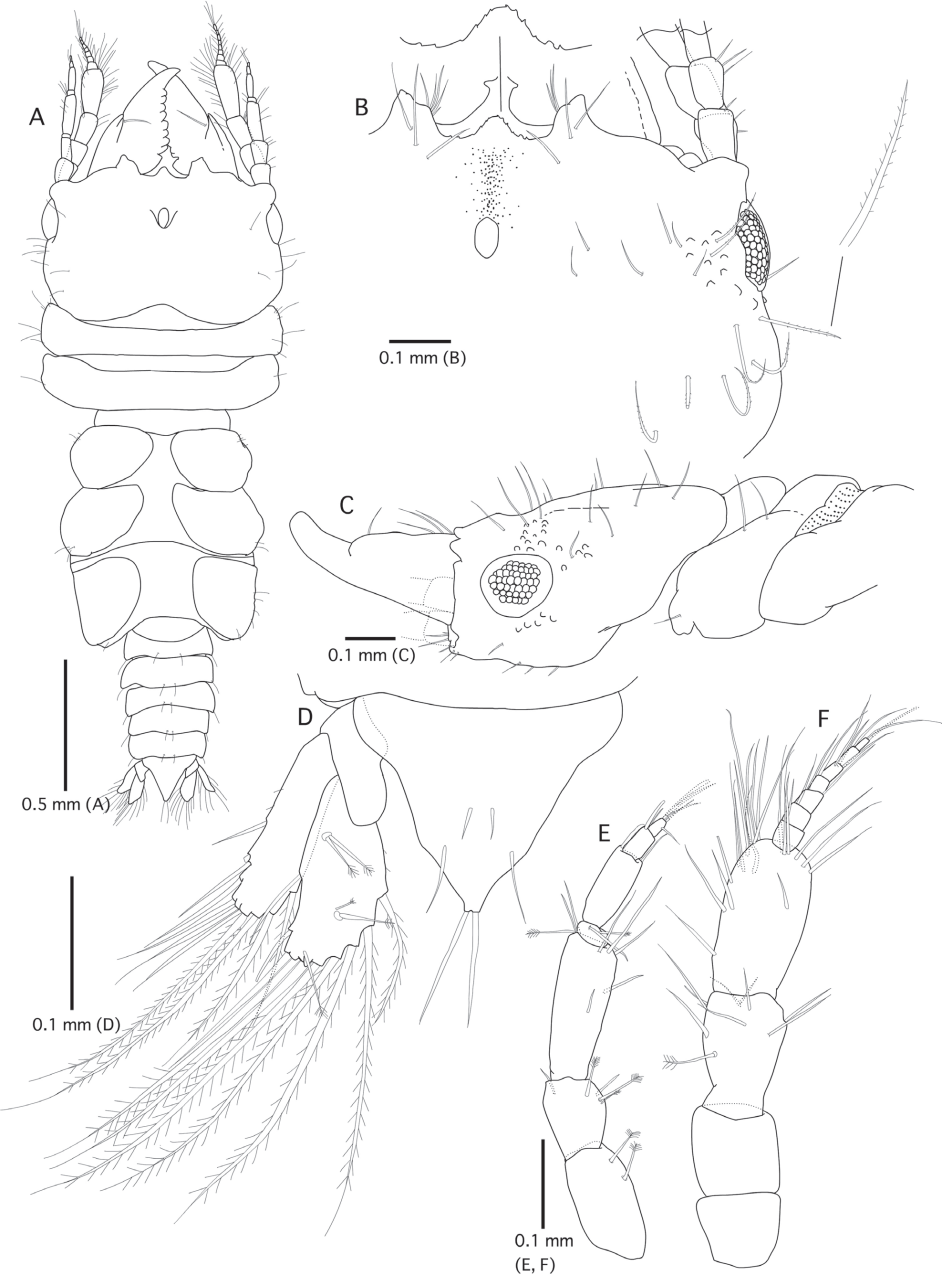
*Gnathiaantennacrassa* sp. nov. (holotype, WAM C-79675) **A** whole body (dorsal view) **B** cephalosome and mandible (dorsal view) **C** pereonite 1, cephalosome, and mandible (left lateral view) **D** pleotelson (dorsal view) **E** left antennula **F** left antenna.

***Body*** (Figs 1, 2A) 2.7 × as long as greatest width, widest at pereonite 2; dorsal surfaces smooth, sparsely setose. ***Cephalosome*** (Fig. 1A–C) rectangular, 0.7 × as long as wide, lateral margins parallel, posterior margin concave; dorsal surface tubercles around eyes; dorsal sulcus wide, shallow, short; translucent region present, elliptical; paraocular ornamentation not developed, posterior median tubercle absent. ***Frontolateral processes*** present. ***Frontal margin*** (Fig. 1B) straight, median point with process. ***External scissura*** present, wide, shallow. ***Mediofrontal process*** present, strong, serrate triangular, without ventral notch and fine setae. ***Superior frontolateral process*** present, single, strong, conical, with three pairs of long simple setae. ***Inferior frontolateral process*** absent. ***Supraocular lobe*** not pronounced; accessory supraocular lobe not pronounced. ***Eyes*** present, round, 0.3 × as long as cephalosome length, contiguous with head surface, ommatidia arranged in rows, eye colour faded.

***Pereon*** (Figs 1, 2A) lateral margins narrowing posteriorly, with few setae; anteriorly smooth. ***Pereonite 1*** not fused dorsally with cephalosome. ***Pereonite 2*** wider than pereonite 1. ***Pereonite 4*** with anterior constriction, median groove present. ***Areae laterales*** present on pereonite 4 and pereonite 5, with two lateral lobes, dorsal sulcus wide. ***Pereonite 6*** with strongly developed lobi laterales, lobuii weak, conical. ***Pereonite 7*** short, narrow, and overlapping pleonite 1. ***Pleon*** epimera not dorsally visible on pleonites. ***Pleonites*** (Figs 1, 2A) lateral margins with one pair of simple setae, with two pairs of simple setae medially. ***Pleotelson*** (Fig. 2D) 0.8 × as long as anterior width, not covered in pectinate scales; lateral margins smooth, anterolateral margins concave, without submarginal seta; posterolateral margin concave, with one pair of submarginal setae, mid-dorsal surface with a pair of sub-median setae, apex with two setae.

***Antennula*** (Fig. 2E) composed of four peduncular and three flagellar articles, 0.8 × as long as antenna; peduncle article 2 0.6 × as long as article 1; article 3 2.1 × as long as article 2, 2.9 × as long as wide; flagellum as long article 3; article 3 with one aesthetasc and one simple seta; article 4 terminating with one aesthetasc and four simple setae. ***Antenna*** (Fig. 2F) composed of four peduncular and seven flagellar articles; maximum width of peduncle article 3.2 × the maximum width of flagellar articles; peduncle article 3 1.7 × as long as wide, 0.7 × as long as article 2, with one penicillate seta, and nine simple setae; article 4 as long as article 3, 1.5 × as long as wide, and with 20 simple setae; flagellum 1.2 × as long as article 4, with seven articles, terminating with two simple setae.

***Mandible*** (Figs 1, 2A–C) 0.7 × as long as cephalosome; triangular, weakly mesially curved; apex 20% total length; mandibular seta present. ***Carina*** present, smooth along proximal half. ***Incisor*** elevated, standing clear of surface. ***Blade*** present, dentate, weakly convex, straight, dentate along 58% of margin. ***Pseudoblade***, internal lobe, and dorsal lobe absent; basal neck short; erisma present; lamina dentata not visible in dorsal view.

**Figure 3.**
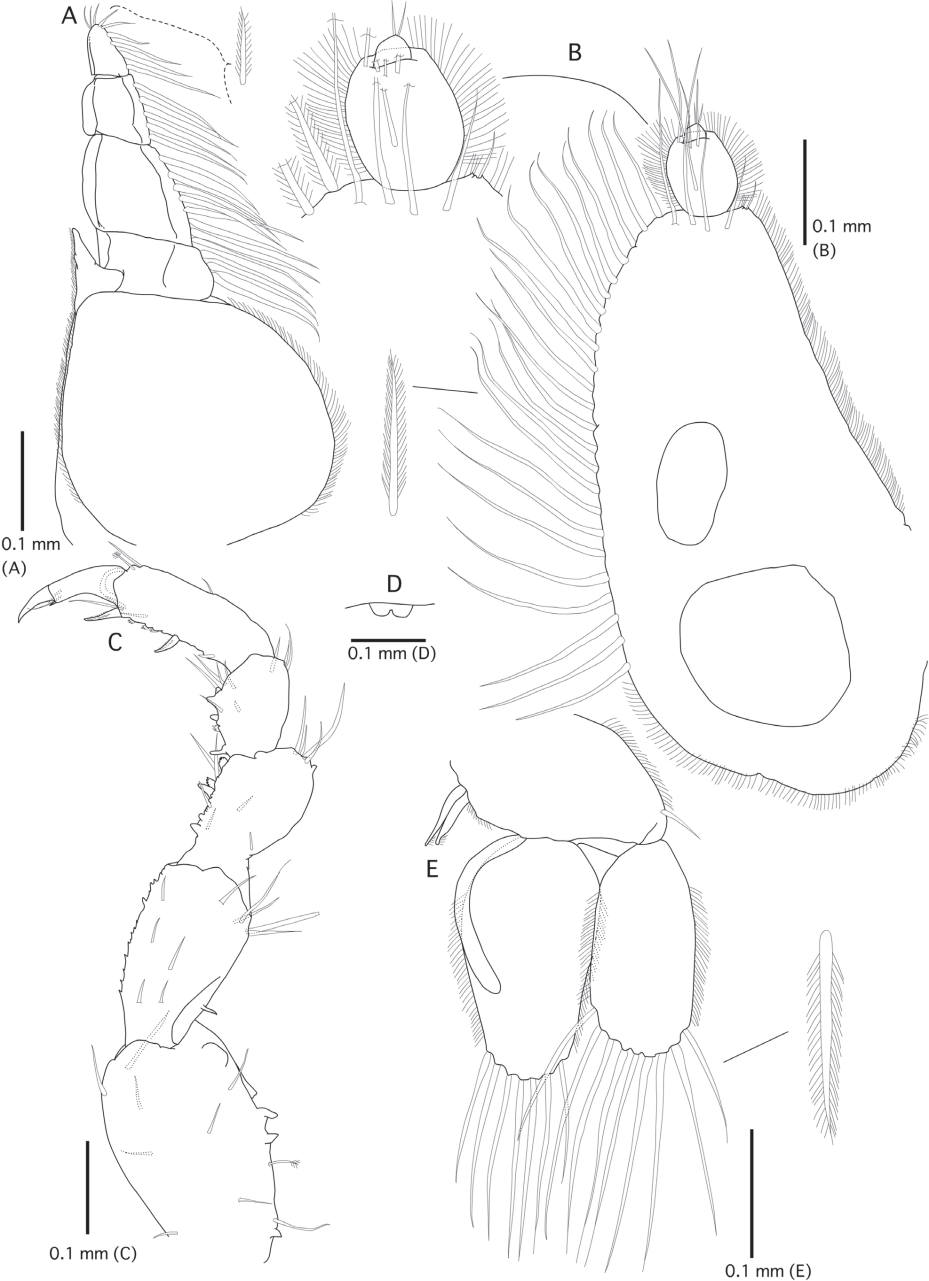
*Gnathiaantennacrassa* sp. nov. (holotype, WAM C-79675) **A** left maxilliped (ventral view) **B** left pylopod (ventral view) **C** left pereopod 2 (lateral view) **D** penes (ventral view) **E** left pleopod 2.

***Maxilliped*** (Fig. 3A). Article 1 lateral margin with continuous marginal scale-setae; article 2 lateral margin with three plumose setae; article 3 lateral margin with eight plumose setae; article 4 lateral margin with five plumose setae; 5 lateral margin with six plumose setae, and three simple setae; endite extending to distal margin of article 2.

***Pylopod*** (Fig. 3B). Article 1 2.0 × as long as wide; with two distinct areolae; without distolateral lobe; posterior and lateral margins forming rounded curve; lateral margin with 24 plumose setae; mesial margin with continuous fringe setae; distal margin with five simple setae; article 2 1.2 × as long as wide, with five simple setae; article 3 semicircular with two short setae.

***Pereopod 2*** (Fig. 3C) sparsely covered with short simple setae on basis and ischium, inferior margins with prominent tubercles on basis to carpus; basis 1.3 × as long as greatest width, superior margin with three simple setae, inferior margin with two simple setae; ischium as long as basis, 2.2 × as long as wide, superior margin with five simple setae; merus 0.4 × as long as ischium, 1.1 × as long as wide, superior margin with four simple setae, and bulbous protrusion, inferior margin with three simple setae; carpus 1.1 × as long as ischium, 0.9 × as long as wide, superior margin with three setae, inferior margin with five setae; propodus 1.6 × as long as ischium, 2.6 × as long as wide, superior margin with two simple setae and one penicillate seta, inferior margin with three pectinate scales, and two robust setae; dactylus 0.4 × as long as propodus. ***Pereopods 3* and *5*** similar proportions of each article as pereopod 2. ***Pereopod 4*** longer than pereopod 2, basis, ischium, and merus slightly longer than those of pereopod 2; propodus somewhat rounded. ***Pereopod 6*** slightly shorter than pereopod 2, basis shorter than that of pereopod 2, distal margin of merus rounded.

***Penes*** (Fig. 3D) with two small papillae, 0.5 × as long as basal width.

***Pleopod 2*** (Fig. 3E). ***Exopod*** 2.2 × as long as wide, distally broadly rounded, with nine plumose setae; ***endopod*** 1.8 × as long as wide, distally narrowly rounded, with seven plumose setae; appendix masculina present, with parallel margins, 0.8 × as long as endopod, distally narrowly rounded; ***peduncle*** 0.5 × as wide as long, mesial margin with two coupling setae, lateral margin with one simple seta. All pleopods similar in shape; exopods each with eight or nine plumose setae; endopods each with 7–9 plumose setae.

***Uropod*** (Fig. 1D). ***Peduncle*** without dorsal setae. ***Uropodal endopod*** 1.6 × as long as greatest width, apex broadly rounded, extending beyond apex of the pleotelson, dorsally with five penicillate setae; lateral and proximomesial margin with seven plumose and three simple setae. ***Uropodal exopod*** not extending to end of endopod, 3.8 × as long as greatest width, apex broadly rounded, reaching almost apex of pleotelson; lateral and proximomesial margin with four plumose and seven simple setae.

#### Distribution.

Known only from the type locality.

#### Habitat of adults.

Sandy substrata of seagrass; 5 m depth.

#### Hosts.

Unknown.

#### Etymology.

The specific name, *antennacrassa*, is derived from Latin, meaning “stout antenna”.

#### Remarks.

Among the other *Gnathia* species worldwide, *G.illepida* Monod, 1923 is similar to *G.antennacrassa* sp. nov., but differs in that the tubercles densely cover the anterior part of the body (cephalosome and pereonites 1–4), the paraocular ornamentation is developed with several distinct tubercles and setae, and the maximum width of peduncle articles of the antenna is 2.4 × of that of the flagellar articles (Monod 1926).

*Gnathiavellosa* Müller, 1988 is also similar, but differ in that tubercles and long setae densely cover the anterior part of the cephalosome and pereonites 2, 3, and anterior part of pereonite 4; the maximum width of peduncle articles of antenna is 2.4 × that of flagellar articles; and three areolae are present on article 1 of the pylopod (Müller 1988).

*Gnathialuxata* Kensley, Schotte & Poore, 2009 differs from our new species as it has three processes on the frontal border but the mesial lobe is present on the mandible and, similarly to *G.vellosa*, it has three areolae present on article 1 of the pylopod (Kensley et al. 2009).

The gnathiid fauna of Western Australia, in contrast to the eastern Australian coast (see Cohen and Poore 1994; Coetzee et al. 2008, 2009; Ferreira et al. 2009, 2010; Farquharson et al. 2012; Svavarsson and Bruce 2012, 2019) remains almost undocumented. Cohen and Poore (1994) mention that *G.mulieraria* Hale, 1924 occurred from Victoria and south Australia to Western Australia. However, the original description mentioned *G.mulieraria* only from South Australia and there is no evidence or reference to its distribution as referred to in Cohen and Poore (1994). Therefore, *G.antennacrassa* represents the first recorded species of Gnathiidae from Western Australia.

### ﻿ Gnathia taurus sp. nov.

Taxon classificationAnimaliaIsopodaGnathiidae

441DEBD3-42B2-56AC-BEFF-0AE7B101F717

https://zoobank.org/450C54D2-99D1-495C-9E66-DA81A9CA3D9F

Figs 4 , 5 , 6

#### Diagnosis.

Large body length more than 8.0 mm; long setae covering most part of dorsal body (cephalosome, pereonites 1–7, and mid-dorsal and lateral parts of pleonites 1–5); frontal margin with rounded mediofrontal process and two superior frontolateral processes; paraocular ornamentation composed of several tubercles and setae; pereonite 1 reaching lateral margins of cephalon, epimera of pleonites 1–5 not prominent; pleotelson 1.3 × longer than its anterior width, eight or nine long setae present on lateral side of pleotelson; mandible almost vertically elongated; article 1 of pylopod with one areolae; appendix masculina of pleopod 2 extending half-length of the endopod; endopod of uropodal rami extends beyond apex of pleotelson; exopod of uropodal rami not extends apex of pleotelson.

#### Material examined.

***Holotype*.** Australia • 1♂ (9.6 mm TL, 8.2 mm BL, dissected); reared from a juvenile collected from a species of *Rhynchobatus* (TL 129 cm, female), Heron Island, Great Barrier Reef (23°26'32.9"S, 151°54' 53.8"E), 7 October 1998. Ian D. Whittington leg. (QM W29819). ***Paratype*.** 1♂ (9.4 mm TL, 8.2 mm BL); same data as holotype (QM W29820).

#### Type locality.

Heron Island, Great Barrier Reef, Australia (23°26'32.9"S, 151°54'53.8"E).

#### Description.

**Figure 4.**
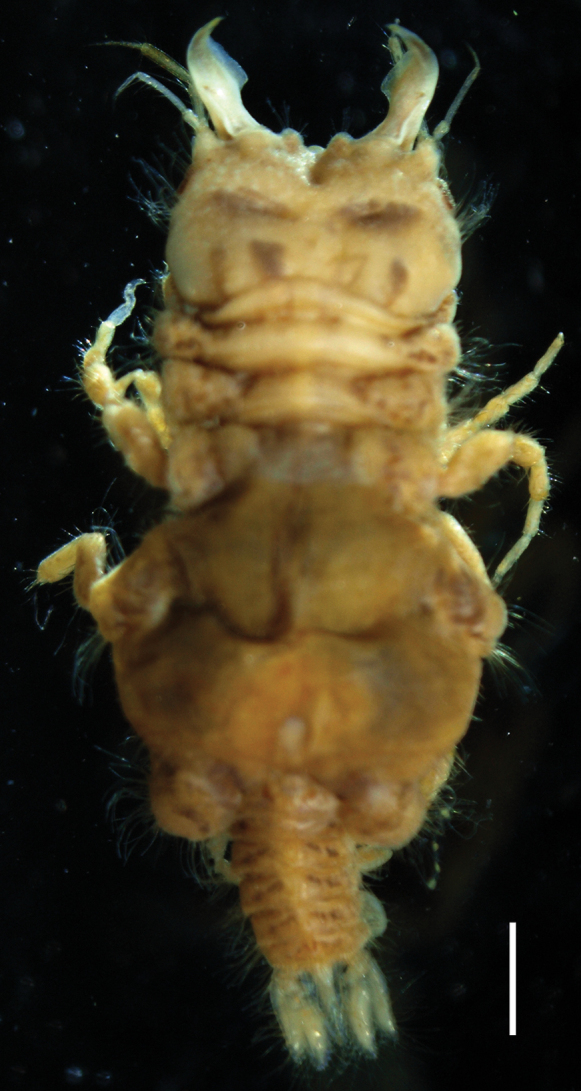
Photograph of fixed *Gnathiataurus* sp. nov. (paratype, QM W29820). Scale bar: 1 mm.

**Figure 5.**
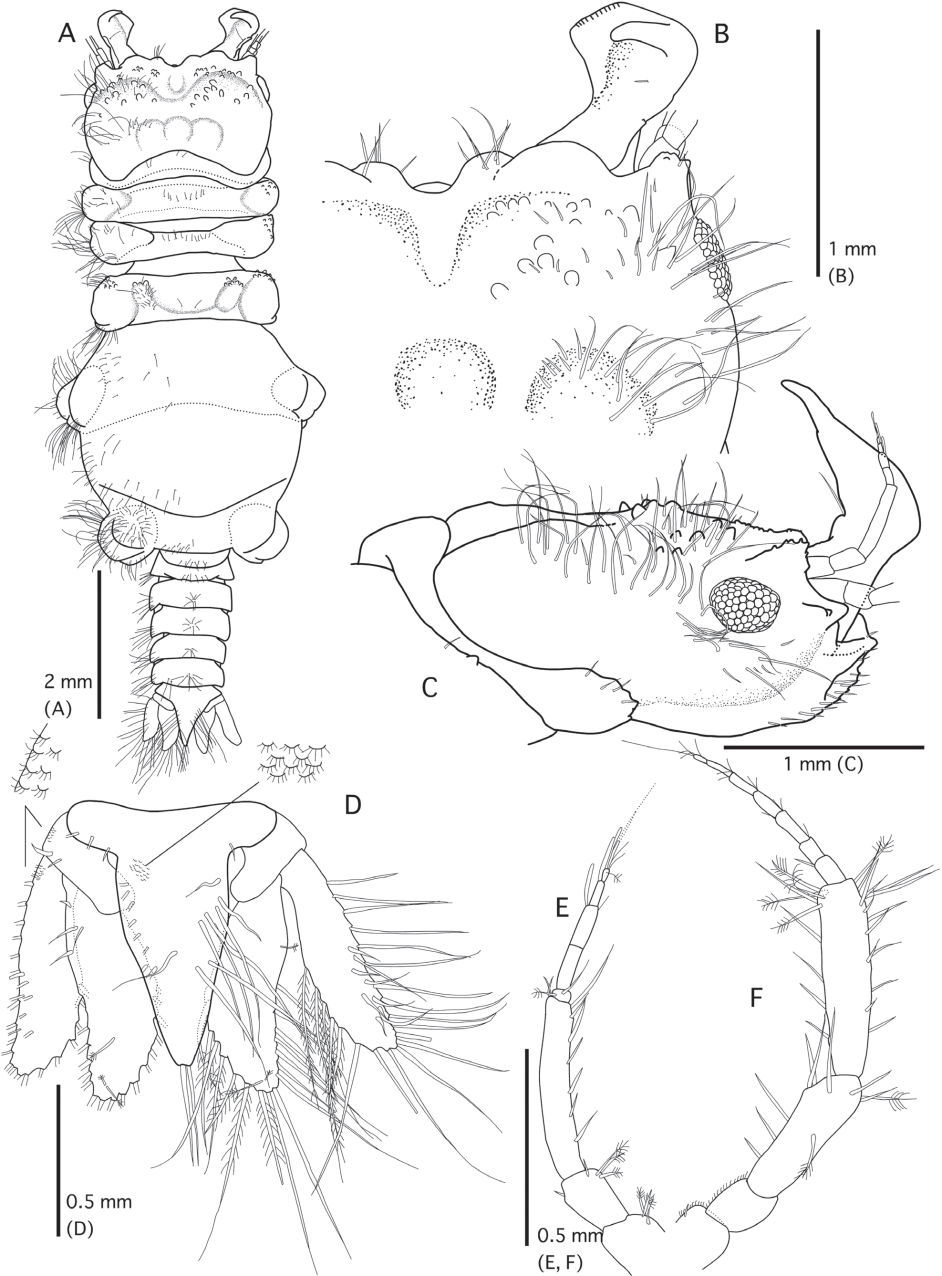
*Gnathiataurus* sp. nov. (holotype QM W29819) **A** whole body (dorsal view) **B** cephalosome and mandible (dorsal view) **C** pereonite 1, cephalosome, and mandible (right lateral view) **D** pleotelson (dorsal view) **E** right antennula **F** right antenna.

***Body*** (Figs 4, 5A) 2.6 × as long as greatest width, widest at pereonite 5; dorsal surfaces with tubercules or granules, densely setose. ***Cephalosome*** (Figs 4, 5A–C) rectangular, 0.7 × as long as wide, lateral margins sub-parallel, posterior margin concave; dorsal surface conspicuous granules anteriorly; dorsal sulcus narrow, shallow, short; translucent region absent; paraocular ornamentation weakly developed and with several tubercles and setae; posterior median tubercle present; lateral tubercles with several long setae. ***Frontolateral processes*** present. ***Frontal margin*** (Fig. 5B) straight and medially concave, median point with process. ***External scissura*** present, wide, shallow. ***Mediofrontal process*** present, weak, rounded, without ventral notch, without setae. ***Superior frontolateral process*** present, single, strong, rounded, with four or five long simple setae. ***Inferior frontolateral process*** absent. ***Supraocular lobe*** not pronounced; accessory supraocular lobe not pronounced. ***Eyes*** present, round, 0.2 × as long as cephalosome length, contiguous with head surface, ommatidia not arranged in rows, eye colour dark brown.

***Pereon*** (Figs 4, 5A) lateral margins ovate, with many setae; with sparse fine granules on anterior parts of pereonites 2–4. ***Pereonite 1*** not fused dorsally with cephalosome; dorsolateral margins not obscured by cephalosome. ***Pereonite 2*** wider than pereonite 1. ***Pereonite 4*** with anterior constriction, median groove absent. ***Areae laterales*** present on pereonite 5, dorsal sulcus wide. ***Pereonite 6*** with strongly developed lobi laterales, lobuii absent. ***Pereonite 7*** short, narrow, and overlapping pleonite 1. ***Pleon*** epimera not dorsally visible on all pleonites. ***Pleonites*** (Figs 4, 5A) lateral margins with 5–7 pairs of simple setae, with 6–9 simple setae medially. ***Pleotelson*** (Fig. 5D) 0.8 × as long as anterior width, covered in pectinate scales; lateral margins smooth, anterolateral margins strongly concave, with 1–3 submarginal setae; posterolateral margin weakly convex, with eight or nine pairs of submarginal setae; mid-dorsal surface with one pair of sub-median setae, apex with two setae.

***Antennula*** (Fig. 5E) composed of four peduncular and four flagellar articles, 0.6 × shorter than antenna; peduncle article 2 1.1 × as long as article 1; article 3 2.2 × as long as article 2, 4.4 × as long as wide; flagellar article 3 with one aesthetasc seta, and one simple seta; article 4 with one aesthetasc seta; article 5 with one penicillate seta, terminating with one aesthetasc seta and three simple setae. ***Antenna*** (Fig. 5F) composed of four peduncular and seven flagellar articles; peduncle article 3 2.8 × as long as wide, 2.5 × as long as article 2, with two penicillate setae, and seven simple setae; article 4 1.3 × as long as article 3, 4.3 × as long as wide, with five penicillate setae, and 18 simple setae; flagellum 0.8 × as long as article 4, terminating with four simple setae.

***Mandible*** (Fig. 5B, C) 0.4 × the head length; strongly curved dorsally; apex positions before dentate blade (but it positions after dentate blade in paratype of Fig. 4), 23% of total length; mandibular seta present. ***Carina*** absent. ***Incisor*** dentate, distal denticulation absent. ***Blade*** present, dentate, straight, proximally convex, dentate for 28% of margin. Pseudoblade, internal lobe and dorsal lobe absent; basal neck long; erisma present; lamina dentata absent.

**Figure 6.**
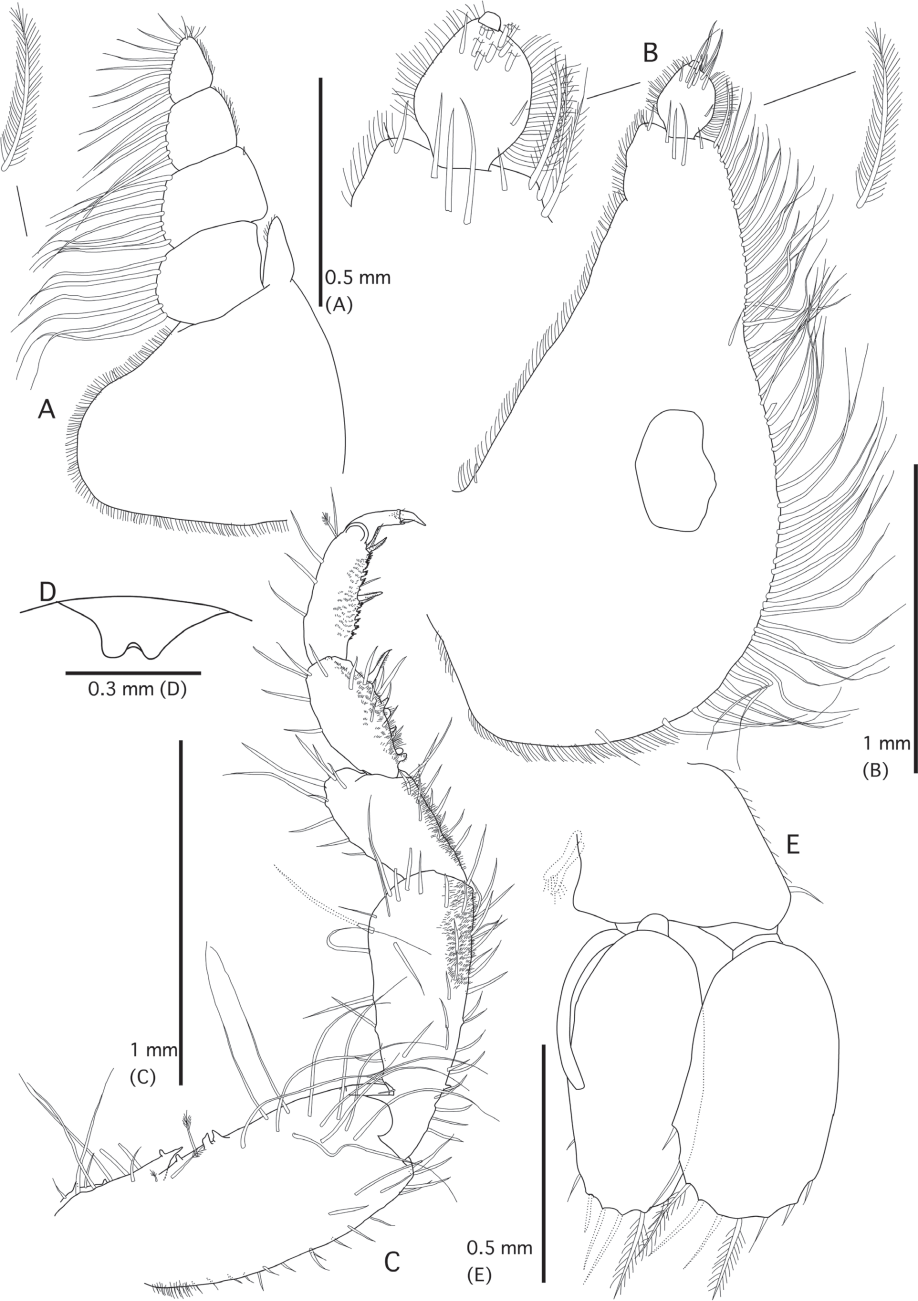
*Gnathiataurus* sp. nov. (holotype QM W29819) **A** right maxilliped (ventral view) **B** right pylopod (ventral view) **C** right pereopod 2 (lateral view) **D** penes (ventral view) **E**. right pleopod 2.

***Maxilliped*** (Fig. 6A). Article 1 lateral margin with continuous marginal scale-setae; article 2 lateral margin with six plumose setae; article 3 lateral margin with seven plumose setae; article 4 lateral margin with six plumose setae; article 5 with nine plumose setae, and six simple setae; endite extending to distal margin of article 2.

***Pylopod*** (Fig. 6B). Article 1 1.9 × as long as wide; with one areola; without distolateral lobe; posterior and lateral margins forming rounded curve; lateral margin with 59 plumose setae; mesial margin with continuous fringe setae; distal margin with five simple setae; article 2 1.3 × as long as wide, with 11 simple setae; article 3 minute and semicircular without setae.

***Pereopod 2*** (Fig. 6C) covered in pectinate scales on inferior margins of ischium, merus carpus, and propodus; basis 2.2 × as long as greatest width, superior margin with 19 simple setae, inferior margin with 21 simple setae; ischium 4.5 × as long as basis, 4.5 × as long as wide, superior margin with 21 simple setae, inferior margin with 11 simple setae; merus 0.3 × as long as ischium, 1.1 × as long as wide, superior margin with eight simple setae and bulbous protrusion, inferior margin with eight simple setae; carpus 1.1 × as long as ischium, 1.9 × as long as wide, superior margin with eight simple setae, inferior margin with four simple setae; propodus 1.1 × as long as ischium, 2.5 × as long as wide, superior margin with three simple setae, superior margin with one penicillate seta, inferior margin with four simple setae, and two denticulate compound spines; dactylus 0.5 × as long as propodus. ***Pereopods 3*, *5*, and *6*** almost same proportion of each article as pereopod 2; basis of pereopod 4 slightly shorter than that of pereopod 2.

***Penes*** (Fig. 6D) produced, penial process 0.4 × as long as basal width.

***Pleopod 2*** (Fig. 6E) ***exopod*** 1.8 × as long as wide, distally broadly rounded, with nine plumose setae; ***endopod*** 2 × as long as wide, distally broadly rounded, with seven plumose setae; appendix masculina present, with parallel margins, 0.5 × as long as endopod, distally bluntly rounded; ***peduncle*** 1.4 × as wide as long, mesial margin with two coupling setae, lateral margin with one simple seta. All pleopods similar in shape; exopods each with 7–11 plumose or simple setae; endopods each with seven or eight plumose or simple setae in total.

***Uropod*** (Fig. 5D). ***Peduncle*** with two dorsal setae. ***Uropodal endopod*** 2.9 × as long as greatest width, apex narrowly rounded, extending beyond apex of pleotelson, dorsally with three penicillate setae; lateral margin weakly convex, lateral margin with nine simple setae; proximomesial margin sinuate, with seven long plumose setae. ***Uropodal exopod*** not extending to end of endopod, apex narrowly rounded, not extending beyond apex of pleotelson, 3.6 × as long as greatest width; lateral margin weakly convex, with 24 simple setae; proximomesial margin weakly convex and sinuate, with five plumose setae.

#### Distribution.

Heron Island, Great Barrier Reef, Australia.

#### Habitat of adults.

Unknown.

#### Hosts.

A species of *Rhynchobatus*. The original data label identified the host as *Rhynchobatusdjiddensis*, but the distribution range of this species is the western Indian Ocean; therefore, the host is most probably *Rhynchobatusaustraliae* Whitley, 1939 or *R.palpebratus* Compagno & Last, 2008, two species that do occur on the GBR (Last et al. 2016).

#### Etymology.

The specific name *taurus*, the second sign of the zodiac, is derived from *taûros*, Latin for bull, and refers to the gnathiid’s dorsally elongated mandible which resemble the horns of a bull.

#### Remarks.

Among *Gnathia* species worldwide, *Gnathiagrandilaris* Coetzee, Smit, Grutter & Davies, 2008 is most similar to *Gnathiataurus* sp. nov., but differs in that its mediofrontal process is acute, the mandible is not vertically elongated, and two areolae are present on article 1 of the pylopod (Coetzee et al. 2008).

*Gnathianubila* Ota & Hirose, 2009 is also similar but the apex of the mediofrontal process is bifid and dentate, the epimera is prominent on pleonites 3–5, and two areolae are present on article 1 of the pylopod (Ota and Hirose 2009b).

### ﻿ Gnathia aff. maculosa

Taxon classificationAnimaliaIsopodaGnathiidae

Ota & Hirose, 2009

4698115F-3733-54AB-8926-CE50572D9AA2

Figs 7A–D , 8

Gnathia maculosa Ota & Hirose, 2009a: 50, 51, 56, 57, figs 1–3, 5.

#### Type locality.

Nakagusku Bay (26°N, 127°E), Okinawajima Island, Japan.

#### Material examined.

Australia • 1♂ (5.0 mm TL, 4.5 mm BL, SEM); reared from a juvenile collected from a cowtail stingray *Pastinachussephen* (Forsskål, 1775) (TL and sex, unknown), Lizard Island, GBR (14°40'08"S, 145°27'34"E), 19 June 1998, Ian D. Whittington leg. (QM W29821). 1♂ (5.5 mm TL, 4.9 mm BL, dissected); reared from a juvenile collected from a species of *Rhynchobatus* (TL 126.5 cm, female), Shark Bay, Heron Island, GBR (23°26'37.03"S, 151°55'5.64"E), 7 Oct. 1998, Ian D. Whittington leg. (QM W29822).

#### Remarks.

**Figure 7.**
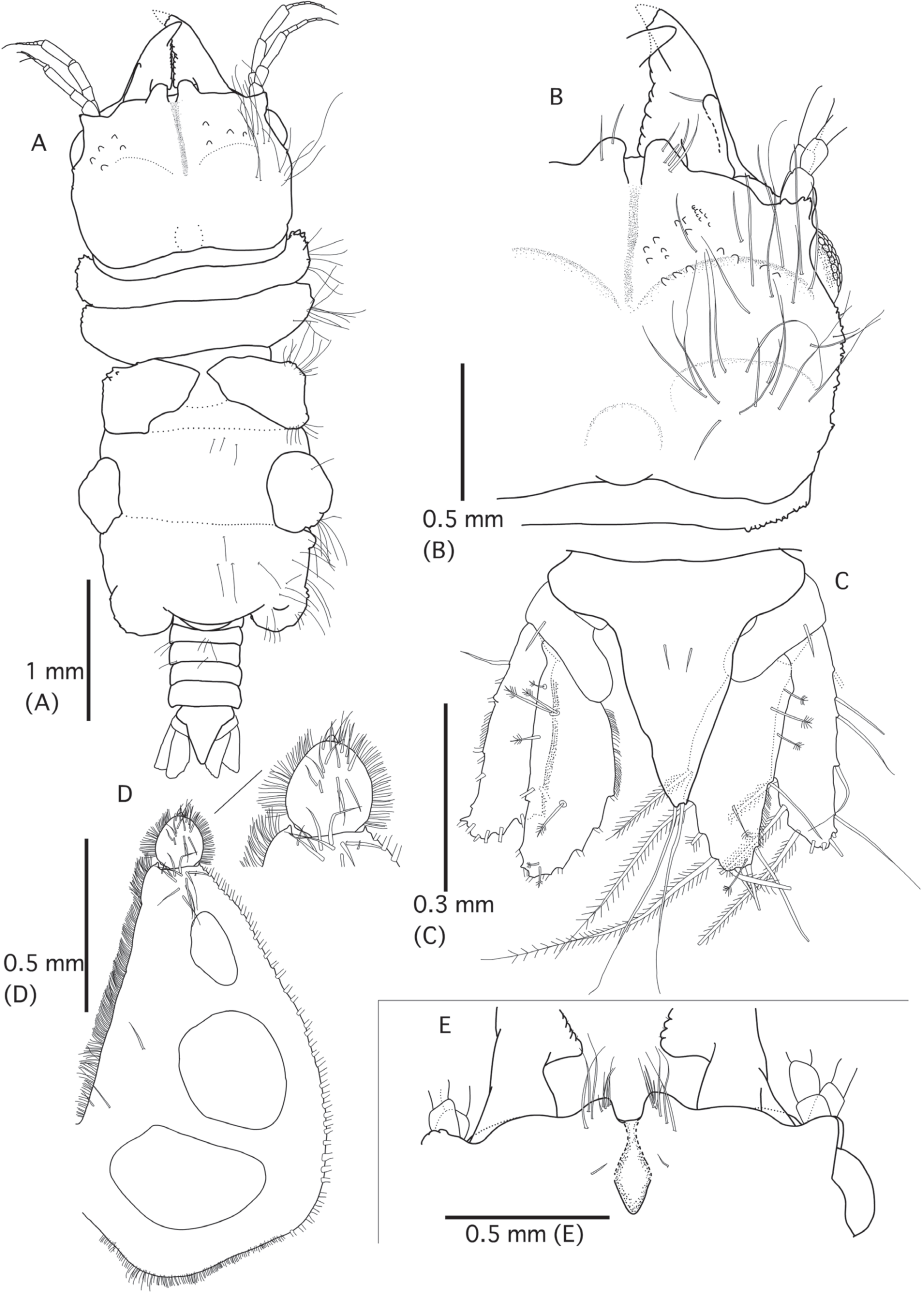
Gnathiaaff.maculosa Ota and Hirose, 2009 (**A–D**; QM W29822) and *G.trimaculata* Coetzee, Smit, Grutter & Davies, 2009 (**E**; QM W29825) **A** whole body (dorsal view) **B** pereonite 1, cephalosome, and mandible (dorsal view) **C** pleotelson (dorsal view) **D** right pylopod (ventral view) **E** frontal border of *G.trimaculata* (dorsal view).

The male morphologies of these GBR specimens show the deep and narrow dorsal sulcus on the cephalosome, the narrow body (Fig. 7A, B), and the almost semicircular pylopod article 1 with three areolae (Fig. 7C). These characters can be identified as *Gnathiamaculosa* Ota & Hirose, 2009. However, this species was originally described from the Ryukyu Islands, southwestern Japan (Ota and Hirose 2009a) and our records are a great distance from this island group. The apices of the frontolateral processes on anterior margin of heads of the present specimens are smooth, while those of original description are serrate (Ota and Hirose 2009a). The number of setae on the pleotelson of the present species is two pairs (Fig. 7D), while that of the original description is three pairs (Ota and Hirose 2009a). Thus, these GBR specimens are identified as G.aff.maculosa.

**Figure 8.**
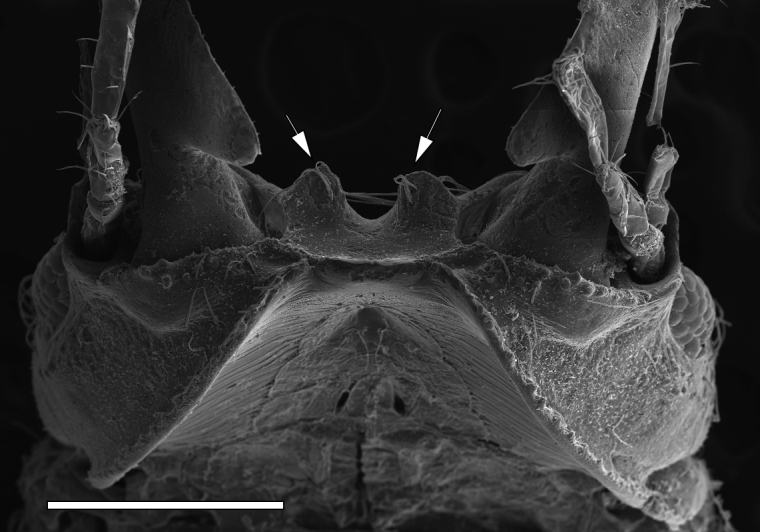
Scanning electron micrograph of the ventral view of the frontal border of Gnathiaaff.maculosa Ota & Hirose, 2009 (QM W29821) showing bundles of several long setae (two arrows). Scale bar: 500 µm.

The GBR specimens of G.aff.maculosa have a bundle of several long setae on the ventral frontal border (Fig. 8). Ota and Hirose (2009a) did not show the ventral frontal border but the Japanese specimens *G.maculosa* also have a bundle of several long setae (YO pers. obs.).

#### Distribution.

Gnathiaaff.maculosa: Lizard Island and Heron Island, Great Barrier Reef, Australia; *Gnathiamaculosa*: Okinawa-jima Island, Kume-jima Island, Ishigaki-jima Island, Japan.

#### Habitat of adults.

Unknown.

#### Hosts.

Two elasmobranch species from GBR: *Pastinachussephen* (Forsskål, 1775), *Rhynchobatus* sp.

#### Hosts of *G.maculosa*.

Ota and Hirose (2009a) recorded the two host elasmobranchs of *G.maculosa* and Ota (2015: table 2) summarised gnathiids ectoparasitising on elasmobranchs in the Ryukyu Islands and documented *G.maculosa* obtained from 11 elasmobranch species including two host species previously recorded by Ota and Hirose (2009a): *Rhynchobatusdjiddensis* (Forsskål, 1775), *Neotrygonorientalis* Last, White & Séret, 2016 [*Neotrygonkuhlii* Müller & Henle, 1841 in Ota 2015], *Taeniurameyeni* Müller & Henle, 1841, *Himanturaundulata* (Bleeker, 1852), *Himantura* sp., *Aetomylaeusvespertilio* (Bleeker, 1852), *Aetobatusocellatus* (Kuhl, 1823) [*Aetobatusnarinari* (Euphrasen, 1790) in Ota 2015], *Rhinopterajavanica* Müller & Henle, 1841, *Nebriusferrugineus* (Lesson, 1831), *Triaenodonobesus* (Rüppell, 1837), and *Negaprionacutidens* (Rüppell, 1837).

#### Site of infection on host in *G.maculosa*.

Gill chambers, interbranchial septa, gill filaments, and the floor of oral cavities. Rarely nostrils, body surface near the gill slits, or claspers.

### ﻿ Gnathia trimaculata

Taxon classificationAnimaliaIsopodaGnathiidae

Coetzee, Smit, Grutter & Davies, 2009

E56E7E63-E0F8-5272-9C65-E62E1AE5B7A1

Fig. 7E

Gnathia trimaculata Coetzee, Smit, Grutter & Davies, 2009: 97, 98, 109–111, figs 1–11.— Ota and Hirose 2009a: 50, 51, figs 4, 5.

#### Type locality.

Off Lizard Island (14°40'54.68"S, 145°26'53.72"E), Australia.

#### Material examined.

Australia • 1♂ (6.4 mm TL, 5.2 BL); reared from a juvenile collected from a cowtail stingray *Pastinachussephen* (Forsskål, 1775) (TL and sex, unknown), Lizard Island, GBR (14°40'54.68"S, 145°26'53.72"E), 19 June 1998, Ian D. Whittington leg. (QM W29823). 2♂ (5.8 mm TL and 4.6 mm BL, 5.7 mm TL and 4.6 mm BL); reared from a juvenile, infested on *P.sephen* (TL and sex, unknown), Heron Island, GBR (23°26'32.9"S, 151°54'53.8"E), 9 July 1998, Ian D. Whittington leg. (QM W29824). 1♂ (4.2 mm TL, 3.6 mm BL, drawings); reared from a juvenile, infested on epaulette shark, *Hemiscylliumocellatum* (Bonnaterre, 1788), Heron Island, GBR (23°26'32.9"S, 151°54'53.8"E), 7 November 1998, Ian D. Whittington leg. (QM W29825).

#### Remarks.

This species can be identified as *Gnathiatrimaculata* Coetzee, Smit, Grutter, & Davies, 2009 by a frontal border with a mediofrontal process divided into two lobes which almost touch anteriorly and form a distinct key-hole shape, four or five pairs of long pappose setae present ventrally on both lobes, a mandible with seven or eight processes on the dentate blade, a cluster of setae between all processes, and an armed carina (Coetzee et al. 2009).

Ota and Hirose (2009a) reported *G.trimaculata* from the Ryukyu Islands, demonstrating a greater number of setae on peduncle 4 of antenna than that of the GBR specimens. In the present material, we observed that the mediofrontal process of our specimens does not almost touch and has a smooth margin (Fig. 7E). Therefore, it appears to be two frontolateral processes instead of one mediofrontal process.

This shape of mediofrontal process looks like that of G.aff.maculosa. Gnathiaaff.maculosa of GBR also has a bundle of several long setae on the ventral frontal border. Thus, these two species cannot be distinguished by the morphology of the frontal border alone. However, *G.trimaculata* can be distinguished from *G.maculosa* by pectinate scales covering the pleotelson, four pairs of long setae on the lateral margin of pleotelson, and a long pear-shaped pylopod with one areola.

This record of *G.trimaculata* establishes two new hosts for this widely distributed species. Ota et al. (2012) recorded *G.trimaculata* from several areas in the Ryukyu Islands and southern Pacific coast of Japan. They demonstrated the first and second stages of the juveniles ectoparasitised four teleost species, while the third stage ectoparasitised 25 elasmobranch species including two unidentified elasmobranch species (see Ota et al. 2012: table 3). Ota (2015: table 2) also showed *G.trimaculata* collected from 18 elasmobranch species including two unidentified elasmobranch species but all of them except for one were already reported by Ota et al. (2012). These host species are listed below; in GBR, our host records of *Pastinachussephen* and *Hemiscylliumocellatum* were not included the previous studies and these are new host records.

#### Distribution.

Off Lizard Island and Heron Island, Great Barrier Reef, Australia. The Ryukyu Islands and southern Pacific coast of Japan.

#### Habitat of adults.

Unknown.

#### Hosts.

Four elasmobranch species from GBR: *Carcharinusmelanopterus* (Quoy & Gaimard, 1824), *Carcharinusamblyrhynchos* (Bleeker, 1856), *Pastinachussephen* (Forsskål, 1775), and epaulette shark *Hemiscylliumocellatum* (Bonnaterre, 1788). Three teleost species from Japan: *Enneapterygiusetheostomus* (Jordan & Snyder, 1902), *Enneapterygiusmiyakensis* Fricke, 1987, *Springerichthysbapturus* (Jordan & Snyder, 1902), 24 elasmobranch species and two unidentified species from Japan: *Urolophusaurantiacus* Müller & Henle, 1841, *Gymnurajaponica* (Temminck & Schlegel, 1850), *Rhynchobatusdjiddensis* (Forsskål, 1775), *Neotrygonorientalis* Last, White & Séret, 2016 [*Neotrygonkuhlii* Müller & Henle, 1841 in Ota et al. 2012 and Ota 2015], *Taeniurameyeni* Müller & Henle, 1841, *Dasyatisizuensis* Nishida & Nakaya, 1988, *Hemitrygonakajei* (Müller & Henle, 1841) [*Dasyatisakajei* (Müller & Henle, 1841) in Ota et al. 2012 and Ota 2015], *Himanturaundulata* (Bleeker, 1852), *Himantura* spp., *Aetomylaeusvespertilio* (Bleeker, 1852), *Aetobatusocellatus* (Kuhl, 1823) [*Aetobatusnarinari* (Euphrasen, 1790) in Ota et al 2012 and Ota 2015], *Aetobatusflagellum* (Bloch & Schneider, 1801), *Rhinopterajavanica* Müller & Henle, 1841, *Mobulamobular* (Bonnaterre, 1788) [*Mobulajapanica* (Müller & Henle, 1841) in Ota et al. 2012 and Ota 2015], *Mobulathurstoni* (Lloyd, 1908) [*Mobuladiabolus* (Shaw, 1804) in Ota et al. 2012 and Ota 2015], *Mobulatarapacana* (Philippi, 1892), *Nebriusferrugineus* (Lesson, 1831), *Rhincodontypus* Smith, 1828, *Stegostomafasciatum* (Hermann, 1783), *Sphyrnalewini* (Griffith & Smith, 1834), *Triaenodonobesus* (Rüppell, 1837), *Negaprionacutidens* (Rüppell, 1837), *Galeocerdocuvier* (Péron & Lesueur, 1822), *Carcharhinusalbimarginatus* (Rüppell, 1837), *Carcharhinuslimbatus* (Müller & Henle, 1839), and *Carcharhinus* spp.

#### Site of infection on host.

Gill chambers, interbranchial septa, gill filaments, and the floor of oral cavities. Rarely nostrils, body surface near the gill slits, or claspers of elasmobranchs. Fins and skin of teleosts.

### ﻿ Gnathia grandilaris

Taxon classificationAnimaliaIsopodaGnathiidae

Coetzee, Smit, Grutter, & Davies, 2008

B7DD0FDC-7DA8-5316-89D9-FE04156A3074

Gnathia grandilaris Coetzee, Smit, Grutter, & Davies, 2008: 608, 613, 614, figs 1–26. —Ota and Hirose 2009b, 43, 44, 51, 54, figs 5–7.

#### Type locality.

Off Lizard Island (14°40'S, 145°27'E), Australia.

#### Material examined.

Australia •1♂ (7.1 mm TL, 6.6 mm BL); reared from a juvenile collected from *P.sephen* (TL and sex, unknown), Heron Island, GBR (23°26'32.9"S, 151°54'53.8"E), 9 July 1998, Ian D. Whittington leg. (QM W29826).

#### Remarks.

The original description of *G.grandilaris* was based on males reared from larvae found infesting a white tip reef shark, *Triaenodonobesus* (Rüppell, 1837), and grey reef sharks, *C.amblyrhynchos*, collected off Lizard Island, GBR (Coetzee et al. 2008) and subsequently reported from the Ryukyu Islands (Ota and Hirose 2009b; Ota 2015). The specimen from Heron Island corresponded well with the original description. This record constitutes a new host and a new locality record for *G.grandilaris*.

#### Distribution.

Lizard Island and Heron Island, Great Barrier Reef, Australia. Okinawa-jima Island, Kume-jima Island, Ishigaki-jima Island, the Ryukyu Islands, Japan.

#### Habitat of adults.

Unknown.

#### Hosts.

Three elasmobranch species from GBR: *Triaenodonobesus* (Rüppell, 1837), *Carcharhinusamblyrhynchos* (Bleeker, 1856), and *Pastinachussephen* (Forsskål, 1775). Seven elasmobranch species from Japan: *Himantura* sp., *Himanturafai* Jordan & Seale, 1906, *Neotrygonorientalis* Last, White & Séret, 2016 [*Neotrygonkuhlii* Müller & Henle, 1841 in Ota and Hirose 2009b and Ota 2015], *Taeniurameyeni* Müller & Henle, 1841, *Mobulajapanica* (Müller & Henle, 1841), *Nebriusferrugineus* (Lesson, 1831), *Triaenodonobesus* (Rüppell, 1837), and *Negaprionacutidens* (Rüppell, 1837).

#### Site of infection on host.

Gill chambers, interbranchial septa, gill filaments, and the floor of oral cavities. Rarely nostrils, body surface near the gill slits, or claspers.

## Supplementary Material

XML Treatment for Gnathia

XML Treatment for Gnathia antennacrassa

XML Treatment for Gnathia taurus

XML Treatment for Gnathia aff. maculosa

XML Treatment for Gnathia trimaculata

XML Treatment for Gnathia grandilaris

## ﻿Additional information

### Author ORCIDs

Yuzo Ota https://orcid.org/0000-0002-7747-6678

Anja Erasmu https://orcid.org/0000-0001-8505-8583

Alexandra S. Grutter https://orcid.org/0000-0003-1688-2821

Nico J. Smit https://orcid.org/0000-0001-7950-193X

### Data availability

All of the data that support the findings of this study are available in the main text.
